# The Influence of Eicosapentaenoic Acid on *Macrobrachium rosenbergii* Broodstock: Ovarian Development, Antioxidant Status, and Lipid Metabolism

**DOI:** 10.1155/anu/3205583

**Published:** 2026-01-15

**Authors:** Yuxin Sun, Hao Chen, Ling Gan, Jiao Liu, Yonghui Jian, Qiyou Xu, Zhili Ding

**Affiliations:** ^1^ College of Life Science, Huzhou University, Zhejiang Provincial Key Laboratory of Aquatic Resources Conservation and Development, Huzhou, 313000, Zhejiang, China, zjhu.edu.cn

**Keywords:** broodstock nutrition, gene expression, lipid source, n-3 highly unsaturated fatty acids, nutritional requirement

## Abstract

Lipids are essential for crustacean reproduction, supporting broodstock growth and ovarian development. However, studies of n‐3 highly unsaturated fatty acids (HUFAs), particularly eicosapentaenoic acid (EPA), on the growth and ovarian development of prawn broodstock remain limited. Accordingly, five experimental diets containing EPA concentrations of 0.12%, 0.79%, 1.46%, 2.21%, and 2.78% were formulated to examine their effects on ovarian development and broodstock health in the giant freshwater prawn *Macrobrachium rosenbergii* (initial weight: 9.32 ± 0.52 g) and to determine dietary EPA requirements during ovarian maturation. The results were as follows: (1) no significant differences in survival rate were observed among groups. Weight gain (WG) initially increased and then declined, reaching the highest values in the 1.46% EPA group, although differences among treatments were not significant. In contrast, hepatopancreas index decreased significantly with increasing dietary EPA (*p* < 0.05). (2) Dietary EPA significantly altered hepatopancreatic fatty acid composition. Saturated fatty acid (SFA) levels showed no significant differences, whereas monounsaturated fatty acids (MUFAs) decreased significantly (*p* < 0.05). In contrast, polyunsaturated fatty acids (PUFAs) and HUFA increased significantly with higher dietary EPA (*p* < 0.05), peaking in the 2.78% EPA group. (3) Antioxidant parameters, including total antioxidant capacity (T‐AOC), total superoxide dismutase (T‐SOD), and glutathione peroxidase (GSH‐Px), followed a pattern of initial increase followed by decline with higher EPA levels (*p* < 0.05). Malondialdehyde (MDA) content showed the opposite trend, reaching its lowest level in the 1.46% EPA group (*p* < 0.05). (4) Ovarian histology revealed that the 1.46% EPA group exhibited a higher proportion of mature oocytes, with most females reaching ovarian development stages III–IV, and this group also showed the highest gonadosomatic index (GSI). Steroid hormone secretion was significantly affected by dietary EPA (*p* < 0.05). (5) At the molecular level, EPA inhibited the expression of genes related to lipid synthesis in the hepatopancreas (*p* < 0.05) and promoted fatty acid *β*‐oxidation, but excessive EPA caused irreversible hepatopancreatic damage. Polynomial regression analysis of steroid hormone secretion indicated that 1.32% and 1.50% dietary EPA supported maximum progesterone (PROG) and 17*β*‐estradiol (E2) levels, respectively. Overall, a dietary EPA level of 1.46% was found to promote broodstock growth, enhance antioxidant capacity, accelerate fatty acid *β*‐oxidation, stimulate steroid hormone secretion, and provide sufficient energy for ovarian development in the giant freshwater prawn.

## 1. Introduction

With the rapid expansion of the aquaculture industry, its production capacity has grown substantially, alongside a growing diversity of farmed species. However, the shortage of high‐quality seedlings has become a critical constraint, emerging as one of the major bottlenecks limiting the sustainable and healthy development of the industry. Against this backdrop, the role of broodstock breeding in aquatic animals has gained unprecedented importance. Recent studies have demonstrated that nutritional factors—including protein, lipids, and carbohydrate—significantly influence broodstock gonadal development [1, 2]. Among these, lipids are indispensable, providing essential fatty acids and playing vital roles in growth, reproduction, and immune regulation [3, 4]. During gonadal development and embryogenesis, lipids and fatty acids not only act as key energy sources but also serve as fundamental components of cellular structures and organelles involved in organ formation [5].

Highly unsaturated fatty acids (HUFAs), defined as polyunsaturated fatty acids (PUFAs) with chain lengths ≥ 20 carbons and three or more double bonds, include eicosapentaenoic acid (C20: 5n‐3, EPA), docosahexaenoic acid (C22: 6n‐3, DHA), and arachidonic acid (C20: 4n‐6, ARA). Among these, EPA and DHA are commonly referred to as n‐3 HUFA [6]. Previous study has shown that when the dietary n‐3 HUFA concentration (6 mg/g) is half that of n‐6 HUFA (12 mg/g), the gonadosomatic index (GSI) and fecundity of mature oriental river prawn (*Macrobrachium nipponense*) are enhanced [7]. Similarly, in Nile tilapia (*Oreochromis niloticus*), diets with linoleic acid/linolenic acid ratios of 3.9 and 0.7 significantly increased egg production per female, so it is suggested that a lower n‐6/n‐3 HUFA ratio is beneficial for upregulating ovarian aromatase expression and improving reproductive performance [8]. Both n‐3 and n‐6 HUFA are essential for the reproduction and growth of Japanese eel (*Anguilla japonica*) broodstock, and dietary fatty acid profiles strongly influence the fatty acid composition of eggs. Notably, an elevated n‐6/n‐3 HUFA ratio has been shown to negatively affect embryonic development [9]. In Chinese tongue sole (*Cynoglossus semilaevis*), the n‐3/n‐6 HUFA ratio regulates spawning performance, egg quality, and larval viability [10]. Similarly, Atlantic halibut (*Hippoglossus hippoglossus*) fed with diets containing 1.8% ARA demonstrated superior spawning performance and larval quality compared to those fed with 0.4% ARA [11]. Furthermore, supplementing ARA during early maturation enhances the final reproductive performance of Pacific white shrimp (*Penaeus vannamei*) [5].

EPA, one of the primary n‐3 HUFA, is an essential nutrient for both humans and aquatic animals [12]. Although research on its specific role in ovarian development remains limited, existing evidence suggests that EPA exerts multiple biological effects. In mammalian models, EPA has been shown to improve immune regulation and display antitumor properties in ovarian cancer [13]. In European eels (*Anguilla anguilla*), interactions between dietary HUFA levels and hormone treatments significantly affect GSI, with females receiving higher levels of EPA and DHA in combination with hormonal induction exhibiting increased GSI [14]. In crustaceans, studies on Chinese shrimp (*Fenneropenaeus chinensis*) broodstock have revealed that egg EPA content is positively correlated with fecundity [15]. Moreover, appropriate dietary supplementation of EPA can promote growth and reproductive development in aquatic animals, whereas both deficiency and excess may impair reproductive performance, inhibit growth, and, in severe cases, lead to mortality [16, 17]. Taken together, these findings indicate that EPA plays a pivotal role in ovarian development. However, EPA requirements vary across species and developmental stages [18], highlighting the importance of determining species‐specific dietary levels for optimal broodstock performance.

The giant freshwater prawn (*Macrobrachium rosenbergii*) is an economically important freshwater prawn in China, valued for both its commercial and ornamental significance. Although previous studies have shown that lipids play a promotive role in ovarian development [19], the precise nutritional requirement for the specific fatty acid EPA during the critical stages of ovarian maturation remains unknown. At present, there are no published reports on the specific EPA requirements of its broodstock during ovarian development. Therefore, this study aims to conduct controlled feeding experiments using diets formulated with graded EPA levels to investigate their effects on broodstock health and ovarian development. Specifically, the study seeks to determine the optimal dietary EPA requirement for its broodstock, elucidate the regulatory mechanisms of EPA in ovarian development, and provide a theoretical foundation for broodstock feed formulation of the giant freshwater prawn.

## 2. Materials and Methods

### 2.1. Experimental Diets

In this experiment, fish meal, soybean meal, chicken meal, and wheat protein meal were used as the primary protein sources, *α*‐starch served as the carbohydrate source, and olive oil together with purified EPA oil (98% purity) was used as the lipid source. Five groups of isonitrogenous and isolipidic diets were formulated, each containing 48% crude protein and 10% crude lipid. The intended EPA supplementation levels were 0%, 0.5%, 1%, 1.5%, and 2%. The actual measured EPA contents in the diets were 0.12%, 0.79%, 1.46%, 2.21%, and 2.78%, respectively; these measured values are used throughout the text to designate the experimental groups. The formulation and proximate composition of the experimental diets are provided in Table 1, and the fatty acid profiles are presented in Table 2.

**Table 1:** Formulation and nutritional composition of experimental diets (%).

Items	Experimental diets				
	0.12%	0.79%	1.46%	2.21%	2.78%
Fish meal	18	18	18	18	18
Soybean meal	16	16	16	16	16
Chicken meal	20	20	20	20	20
Wheat gluten meal	18	18	18	18	18
*α*‐starch	15	15	15	15	15
Olive oil	3	2.6	2.1	1.6	1.1
EPA purified oil	0	0.4	0.9	1.4	1.9
Soy lecithin	1	1	1	1	1
Cholesterol	0.5	0.5	0.5	0.5	0.5
Vitamin premix^a^	0.5	0.5	0.5	0.5	0.5
Mineral premix^b^	0.5	0.5	0.5	0.5	0.5
Choline chloride	0.5	0.5	0.5	0.5	0.5
Vitamin C	0.05	0.05	0.05	0.05	0.05
Antioxidant	0.05	0.05	0.05	0.05	0.05
Calcium dihydrogen phosphate	1.5	1.5	1.5	1.5	1.5
Sodium carboxymethyl cellulose	2	2	2	2	2
Cellulose	3.4	3.4	3.4	3.4	3.4
Total	100	100	100	100	100
Proximate composition Moisture (%)	5.68	5.45	5.77	6.03	5.21
Crude protein (%)	48.37	48.70	48.60	48.95	48.98
Crude lipid (%)	10.09	10.45	10.32	10.13	10.27
Ash (%)	11.88	11.44	10.95	11.50	10.50
Gross energy (kJ/g)	14.99	15.18	15.18	15.00	15.34

^a^Vitamin premix contained the following (g/kg): VA 6.4 g, VC 151.52 g, VE 72 g, VD_3_ 0.8 g, VK 32 g, VB_1_ 3.2 g, VB_2_ 9 g, VB_6_ 4 g, VB_12_ 0.08 g, nicotinic acid 16 g, folic acid 1 g, inositol 64 g, biotin 0.2 g, calcium pantothenate 14 g, zeolite powder 777.02 g.

^b^Mineral premix contained the following (g/kg): CuSO_4_·5H_2_O 1.95 g, ZnSO_4_·7H_2_O 30.91 g, MnSO_4_·H_2_O 5.23 g, FeSO_4_·7H_2_O 24.83 g, Ca(IO3)_2_ 0.46 g, Na_2_SeO_3_ 0.09 g, CoCl_2_·6H_2_O 0.32 g, MgSO_4_·7H_2_O 244.9 g.

**Table 2:** Fatty acid composition of the experimental diets.

^a^Fatty acid	Experimental diets				
	0.12%	0.79%	1.46%	2.21%	2.78%
C14: 0	0.14	0.12	0.11	0.11	0.11
C16: 0	1.79	1.66	1.64	1.57	1.42
C16: 1	0.17	0.18	0.16	0.15	0.16
C18: 0	0.67	0.51	0.60	0.62	0.43
C18: 1n9	3.11	2.90	2.49	2.10	1.95
C18: 2n6	2.59	2.57	2.34	2.11	2.05
C20: 0	0.04	0.03	0.03	0.03	0.02
C18: 3n3	0.36	0.35	0.31	0.27	0.25
C20: 1	0.08	0.07	0.07	0.06	0.05
C20: 4n6	0.08	0.08	0.08	0.08	0.08
C20: 5n3	0.12	0.79	1.46	2.21	2.78
C22: 6n3	0.28	0.30	0.26	0.26	0.26
^b^ *Σ*SFA	2.84	2.47	2.55	2.49	2.11
^c^ *Σ*MUFA	3.50	3.27	2.82	2.40	2.26
^d^ *Σ*PUFA	3.54	4.21	4.58	5.07	5.58
^e^ *Σ*HUFA	0.48	1.20	1.84	2.61	3.20
^f^ *Σ*n‐3	0.79	1.48	2.08	2.81	3.38
^g^ *Σ*n‐6	2.75	2.73	2.50	2.26	2.20
n‐3/n‐6	0.29	0.54	0.83	1.25	1.53

^a^Not all fatty acid components analyzed are included in this table, which shows the major fatty acids. Some fatty acids that are present in minor amounts, trace amounts, or were not detected are not listed in the table. The fatty acid composition in the table is expressed as the average of the two measurements.

^b^ *Σ*SFA (total saturated fatty acid) = C12: 0 + C14: 0 + C16: 0 + C18: 0 + C20: 0 + C22: 0

^c^ *Σ*MUFA (total monounsaturated fatty acid) = C16: 1 + C18: 1n‐9 + C20: 1n‐9 + C22: 1n‐9

^d^ *Σ*PUFA (total polyunsaturated fatty acid) = C18: 2n‐6 + C18: 3n‐3 + C18: 3n‐6 + C20: 2 + C20: 3n‐6 + C20: 3n‐3 + C20: 4n‐6 + C20: 5n‐3 + C22: 6n‐3

^e^ *Σ*HUFA (total highly unsaturated fatty acid) = C20: 4n‐6 + C20: 5n‐3 + C22: 6n‐3

^f^ *Σ*n‐3 (total n‐3 series of fatty acids) = C18: 3n‐3 + C20: 3n‐3 + C20: 5n‐3 + C22: 6n‐3

^g^ *Σ*n‐6 (total n‐6 series of fatty acids) = C18: 2n‐6 + C18: 3n‐6 + C20: 3n‐6 + C20: 4n‐6.

### 2.2. Experimental Animal Rearing and Management

The broodstock used in this experiment were supplied by Zhejiang Zhongyi Aquatic Seedling Co., Ltd. and reared for 56 days at an aquaculture facility in Changxing County, Zhejiang Province. Prior to the experiment, 600 healthy giant freshwater prawns with robust body condition and an average initial weight of 9.32 ± 0.52 g were selected and randomly assigned to five dietary groups, each with four replicates (30 prawns per replicate). The prawns, maintained at a male‐to‐female ratio of 2:1, were acclimated for 3 days in disinfected cement tanks (1.5 × 1 × 1 m) before the feeding trial commenced.

During the rearing period, prawns were fed experimental diets at 1.5%–2.0% of body weight per day, with rations adjusted biweekly. Feeding was conducted twice daily, at 08:00 and 16:30. Feeding behavior was monitored daily, mortalities were recorded, and uneaten feed was promptly removed to minimize water quality deterioration. To maintain suitable culture conditions, one‐third of the tank water was replaced every 2 days. Key water quality parameters were maintained as follows: temperature 23.8–30.8°C, dissolved oxygen 5–6 mg/L, pH 7.8–8.4, and ammonia‐nitrogen ≤ 0.3 mg/L.

### 2.3. Sampling Collection and Calculations

At the end of the 56‐day feeding trial, the giant freshwater prawn broodstocks were fasted for 24 h prior to sampling. The number of surviving individuals in each group was recorded, and body weight and body length were measured. Female prawns were placed on ice trays to determine gonadal development stage, and individuals at the same stage were pooled for sampling.

Hemolymph was collected from the pericardial cavity using a sterile syringe inserted behind the carapace. For each tank, samples from three prawns at the same gonadal stage per tank were pooled, kept on ice, centrifuged at 3500 *r*/min for 20 min, and the supernatant was stored at −80°C for subsequent analyses. The hepatopancreas and gonads were carefully excised, weighed, and immediately preserved in labeled Eppendorf tubes at −80°C for fatty acid composition, antioxidant capacity, and gene expression analyses. In addition, hepatopancreas and ovary tissues from one randomly selected prawn per tank were fixed in 4% paraformaldehyde for histological preparation. The following indices were calculated:

SRsurviving rate,%= Final number of prawns/Initial number of prawns×100.

WGweight gain,%=Final weight− Initial weight/ Initial weight×100.

SGRspecific growth rate,%/d=Ln final weight− Ln initial weight/ Breeding days×100.

HSIhepatosomatic index,%=Wet hepatopancreas weight/Wet body weight×100.

GSIgonadosomatic index,%= Wet ovary weight/Wet body weight×100.

### 2.4. Methods for Sample Analysis

#### 2.4.1. Determination of Nutritional Components in Feed

The proximate composition of the experimental diets was analyzed following standard procedures (AOAC, 2005), with three replicates per diet. Feed samples were oven‐dried at 105°C to constant weight to determine moisture content. Crude lipid was extracted and quantified using a Soxhlet apparatus with anhydrous diethyl ether as the solvent. Crude protein content was determined using the Dumas combustion method, with nitrogen quantified by a thermal conductivity detector. The determination of gross energy (GE) in feed followed the national standard (China National Standard, 2024), and a bomb calorimeter (Model C2000, IKA Group, Staufen, Germany) was used for the analysis. Ash content was determined after combustion at 550°C for 6 h in a muffle furnace.

#### 2.4.2. Determination of Fatty Acids

The fatty acid composition of the diets and hepatopancreas was determined using gas chromatography–mass spectrometry (GC–MS; 8890B‐7693A, Agilent, USA) equipped with a CD‐2560 capillary column (100 m × 0.25 mm × 0.25 μm; Anpel Laboratory Technologies, Shanghai). Analyses were performed according to the procedures established by Qingdao Youfeite Testing Co., Ltd.

#### 2.4.3. Determination of Antioxidant Capacity

Total antioxidant capacity (T‐AOC), total superoxide dismutase (T‐SOD), and glutathione peroxidase (GSH‐Px) activities, as well as malondialdehyde (MDA) content, were determined using commercial kits (Nanjing Jiancheng Bioengineering Institute, Nanjing, China). All assays were conducted following the manufacturer’s instructions and relevant literature, and absorbance was measured with a full‐wavelength microplate reader (Thermo Scientific Multiskan GO 1510).

#### 2.4.4. Determination of Sex Steroid Hormones

The serum levels of estradiol (E2), progesterone (PROG), and testosterone (T) in *M. rosenbergii* were quantified using a double‐antibody sandwich enzyme‐linked immunosorbent assay (ELISA) with commercial kits (Jiangsu Enzyme‐linked Biotechnology Co., Ltd., China). The procedure involved: (1) coating microplates with purified antibodies specific for E2, PROG, and T; (2) sequential addition of standards or test samples together with HRP‐labeled detection antibodies to form antibody–antigen–enzyme–antibody complexes; and (3) colorimetric detection using TMB substrate, with absorbance measured at 450 nm using a microplate reader.

#### 2.4.5. Gene Expression Analysis

For gene analysis, total RNA was extracted from the hepatopancreas samples using the Trizol reagent. Using agarose gel electrophoresis and the Thermo NanoDrop 2000 (Thermo Fisher Scientific [China] Ltd., China), the concentration and quality of total RNA were evaluated. Subsequently, a reverse transcription kit (Takara, Japan) was used to transcribe the total RNA into cDNA. Through the use of quantitative real‐time polymerase chain reaction (qRT‐PCR), the mRNA expression of genes associated with lipid metabolism in the hepatopancreas was investigated. The primers for qRT‐PCR were designed with the Primer 5 software based on sequences from the NCBI, and then Sangon Biotech Co., Ltd. (Shanghai, China) synthesized the primers (Table 3).

**Table 3:** Primer sequences used for qRT‐PCR.

Gene	Primer sequences (5’‐3’)	GenBank	Product length (bp)
*β*‐actin	F:TCCGTAAGGACCTGTATGCC R:TCGGGAGGTGCGATGATTTT	AY651918.2	136
*fas*	F:TCACTTCTCAACACCCAATCCA R:TTGCAGACCGAAGAAGGACG	XM_067091759.1	152
*acc*	F:GATGAGGGATTCAAGCCCAGTT R:TCCCTGTCTTCACCCCACGA	XM_067090789.1	158
*srebp-1*	F:TGTGTCATAGGGTTGACCGC R:ATGGACACTGCAGGTATCGC	XM_067126347.1	92
*cpt-1*	F:AGATTGCCTCTGCCTGC R:AAAGCAGCGTGGGTGAC	XM_067095934.1	164
*acox-1*	F:TTGACAGCGTCGAAACCGTA R:TCTCAAGGACGCCAACAGAC	XM_067089519.1	185
*ampkα*	F:TGGAAAGTGAGCATTGACGAAG R:CATTGGGGTCACGCAACAGA	XM_067097582.1	121
*fabp-3*	F:AACGACGAATGGACGCTGAA R:TTCCCTTAGTGGCGTTCTGG	XM_067082281.1	169
*pparγ*	F:CCTACCGTCCAGAGGCTATG R:AAGGACTGTGGCCATGATCA	XM_067110732.1	150
*scd*	F:CTGCTGTCGTCACTGCTTGCTT R:ACTCGTCGTCCAATCATCTCAGGT	XM_067098533.1	391

*Note:* fatty acid synthase (*fas*), acetyl‐CoA carboxylase (*acc*), sterol regulatory element‐binding protein‐1 (*srebp-1*), acyl‐CoA oxidase‐1 (*acox-1*), AMP‐activated protein kinase alpha (*ampkα*), carnitine palmitoyltransferase‐1 (*cpt-1*), fatty acid binding protein‐3 (*fabp-3*), stearoyl‐CoA desaturase (*scd*), and peroxisome proliferator‐activated receptor gamma (*pparγ*) after amplification.

A Fast SYBR Mixture (Cwbio Co., Ltd., China) was used in the CFX96TM Touch Real‐Time PCR System (Bio‐Rad Laboratories, Inc., USA) for the qRT‐PCR analysis. The PCR conditions were as follows: 10 min at 95°C, 40 cycles of 10 s at 95°C, 30 s at 58°C, and 32 s at 72°C. Following qRT‐PCR, the melting curve was plotted to verify the amplified product’s specificity within a temperature range from 65 to 95°C with a ramp rate of 0.5°C/5 s. Due to its stable expression, *β*‐actin was chosen as the reference gene to standardize our samples in this investigation. The 2^−△△Ct^ method was used to calculate the transcripts of fatty acid synthase (*fas*), acetyl‐CoA carboxylase (*acc*), sterol regulatory element‐binding protein‐1 (*srebp-1*), acyl‐CoA oxidase‐1 (*acox-1*), AMP‐activated protein kinase alpha (*ampkα*), carnitine palmitoyltransferase‐1 (*cpt-1*), fatty acid binding protein‐3 (*fabp-3*), stearoyl‐CoA desaturase (*scd*), and peroxisome proliferator‐activated receptor gamma (*pparγ*) after amplification. Each sample was subjected to three independent biological replicates.

#### 2.4.6. Histological Analysis of Hepatopancreas and Ovary

Hepatopancreas and ovary samples were fixed in 4% paraformaldehyde for 24 h and subsequently transferred to 70% ethanol. Tissues were then dehydrated, embedded in paraffin, and sectioned at 5 μm thickness. The sections were stained with hematoxylin–eosin (HE) and scanned by Hangzhou Hawk Biological Co., Ltd. Oocyte diameters were measured using the software provided by the company.

### 2.5. Data Analysis

All experimental data were first assessed for normality and homogeneity of variance. One‐way analysis of variance (ANOVA) was conducted to compare differences among groups, followed by Tukey’s post hoc test for multiple comparisons. Statistical significance was defined at *p*  < 0.05. Data are presented as mean ± standard deviation (mean ± SD). All statistical analyses were performed using SPSS 26.0 software.

## 3. Results

### 3.1. Effect of Different EPA Levels on the Growth Performance of *M. rosenbergii* Broodstock

The growth performance of the giant freshwater prawn broodstock under different dietary EPA levels is summarized in Table 4. No significant differences were observed in initial body weight or survival rate among the experimental groups. Final body weight, weight gain (WG), and specific growth rate (SGR) exhibited a trend of increasing initially and then decreasing, with the highest values recorded in the 1.46% EPA group; however, these differences were not statistically significant among groups. In contrast, the hepatosomatic index (HSI) decreased significantly with increasing dietary EPA concentration, reaching its lowest value in the 2.78% EPA group (*p* < 0.05).

**Table 4:** Effect of different EPA levels on the growth performance of *M. rosenbergii* broodstock.

Groups	0.12%	0.79%	1.46%	2.21%	2.78%
IBW (g)	9.33 ± 0.31	9.38 ± 0.23	9.20 ± 0.90	9.11 ± 0.79	9.57 ± 0.38
FBW (g)	21.05 ± 0.64	22.23 ± 1.05	22.85 ± 1.05	21.57 ± 1.01	21.71 ± 0.29
SR (%)	73.33 ± 10.00	73.33 ± 8.82	75.56 ± 8.39	72.22 ± 1.92	73.33 ± 6.67
WG (%)	125.87 ± 14.67	137.02 ± 9.39	150.10 ± 29.19	137.49 ± 11.81	127.03 ± 8.39
SGR (%/d)	1.45 ± 0.11	1.54 ± 0.08	1.63 ± 0.21	1.54 ± 0.09	1.46 ± 0.07
HSI (%)	8.63 ± 0.49^a^	7.85 ± 0.14^ab^	7.54 ± 0.33^b^	7.27 ± 0.13^b^	7.12 ± 0.52^b^

*Note:* Values (mean ± SD; *n* = 3) with the different superscripts are significantly different (*p* < 0.05).

Abbreviations: FBW, final body weight; HSI, hepatosomatic index; IBW, initial body weight; SGR, specific growth rate; SR, survival rate; WG, weight gain.

### 3.2. Effect of Different EPA Levels on Fatty Acid Composition of Hepatopancreas in *M. rosenbergii* Broodstock

The influence of dietary EPA levels on the hepatopancreas lipid profile of the giant freshwater prawn broodstock is presented in Table 5. As dietary EPA increased, the content of saturated fatty acids (SFAs) in the hepatopancreas did not differ significantly among groups, whereas monounsaturated fatty acids (MUFAs) decreased significantly (*p* < 0.05). Conversely, higher EPA supplementation significantly elevated the levels of PUFAs and HUFAs in the hepatopancreas, with the maximum values observed in the 2.78% EPA group (*p* < 0.05).

**Table 5:** Effect of different EPA levels on fatty acid composition of hepatopancreas in *M. rosenbergii* broodstock.

Fatty acid	Experimental diets				
	0.12%	0.79%	1.46%	2.21%	2.78%
C14: 0	2.65 ± 0.20	2.47 ± 0.22	2.76 ± 0.06	2.66 ± 0.11	2.67 ± 0.13
C16: 0	19.27 ± 1.08	18.63 ± 0.47	18.10 ± 0.96	19.13 ± 1.72	18.93 ± 0.76
C16: 1	4.19 ± 1.14	2.45 ± 0.07	3.61 ± 0.61	2.68 ± 0.70	2.87 ± 0.39
C18: 0	7.71 ± 0.17^b^	7.56 ± 0.72^b^	8.13 ± 0.46^ab^	8.52 ± 0.10^ab^	9.26 ± 0.66^a^
C18: 1n9	38.43 ± 1.54^a^	37.73 ± 0.92^a^	36.00 ± 0.40^a^	32.27 ± 0.72^b^	30.80 ± 1.70^b^
C18: 2n6	16.33 ± 0.72	17.67 ± 1.22	16.83 ± 1.40	17.87 ± 0.65	17.23 ± 0.49
C18: 3n3	1.62 ± 0.04^a^	1.52 ± 0.09^ab^	1.40 ± 0.11^bc^	1.35 ± 0.08^bc^	1.27 ± 0.01^c^
C20: 1	1.19 ± 0.08^b^	1.18 ± 0.09^b^	1.54 ± 0.09^a^	1.20 ± 0.01^b^	1.48 ± 0.12^a^
C20: 4n6	0.68 ± 0.05^a^	0.81 ± 0.01^a^	0.50 ± 0.05^b^	0.67 ± 0.07^a^	0.54 ± 0.06^b^
C20: 5n3	1.43 ± 0.69^c^	3.39 ± 0.36^bc^	4.25 ± 0.31^b^	6.69 ± 0.91^a^	7.66 ± 1.26^a^
C22: 6n3	2.26 ± 0.67	2.28 ± 0.45	1.67 ± 0.10	2.00 ± 0.44	1.75 ± 0.34
*Σ*SFA	31.40 ± 1.18	30.76 ± 0.41	30.86 ± 1.38	32.48 ± 1.78	33.05 ± 1.25
*Σ*MUFA	44.28 ± 1.58^a^	41.74 ± 0.94^a^	41.66 ± 1.04^a^	36.61 ± 0.52^b^	35.67 ± 2.16^b^
*Σ*PUFA	24.06 ± 2.27^b^	27.30 ± 0.84^ab^	27.08 ± 2.05^ab^	30.63 ± 1.87^a^	31.05 ± 1.77^a^
*Σ*HUFA	4.64 ± 1.80^c^	6.48 ± 0.80^abc^	6.42 ±0.41^bc^	9.35 ± 1.42^ab^	9.95 ± 1.61^a^
*Σ* n‐3	5.58 ± 1.36^c^	7.42 ± 0.73^bc^	7.73 ± 0.55^bc^	10.32 ± 1.32^ab^	11.05 ± 1.57^a^
*Σ* n‐6	17.38 ± 1.02	18.65 ± 1.27	17.54 ± 1.45	18.72 ± 0.65	17.99 ± 0.49

*Note:* Not all fatty acid components analyzed are included in this table, which shows the major fatty acids. Some fatty acids that are present in minor amounts, trace amounts, or were not detected are not listed in the table. The computational methodology of *Σ*SFA, *Σ*MUFA, *Σ*PUFA, *Σ*HUFA, *Σ*n‐3, and *Σ*n‐6 is the same as in Table 2. Values (mean ± SD; *n* = 3) with the different superscripts are significantly different (*p* < 0.05).

Specifically, the contents of oleic acid and linolenic acid declined significantly with increasing dietary EPA, while stearic acid content increased (*p* < 0.05). Moreover, n‐3 fatty acid levels in the 2.78% EPA group were significantly higher than those in the 0.12%, 0.79%, and 1.46% EPA groups (*p* < 0.05), whereas n‐6 fatty acid levels were not significantly affected by dietary EPA concentration.

### 3.3. Effect of Different EPA Levels on Antioxidant Capacity in *M. rosenbergii* Broodstock

The effects of dietary EPA levels on hepatopancreas antioxidant enzyme activities and MDA content are presented in Figure 1. With increasing EPA concentration, T‐AOC initially increased and then decreased, reaching its maximum in the 1.46% EPA group (*p* < 0.05). Similarly, the activities of T‐SOD and GSH‐Px in the hepatopancreas exhibited comparable trends, peaking in the 1.46% and 0.79% EPA groups, respectively (*p* < 0.05). In contrast, MDA content displayed a pattern of first decreasing and then increasing with higher EPA levels, with the 0.79% and 1.46% EPA groups showing significantly lower MDA levels compared to the other groups (*p*  < 0.05).

Figure 1The antioxidant capacity of the hepatopancreas in female *M. rosenbergii* broodstock. (A) Total antioxidant capacity (T‐ AOC), (B) total superoxide dismutase (T‐SOD), (C) glutathione peroxidase (GSH‐Px), and (D) malondialdehyde (MDA). Data are mean ± SD (*n* = 3). Different letters above bar indicate significant difference (*p* < 0.05).

**Figure:**
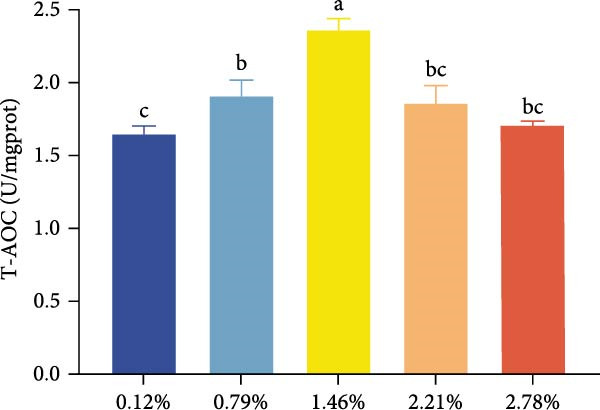
(A)

**Figure:**
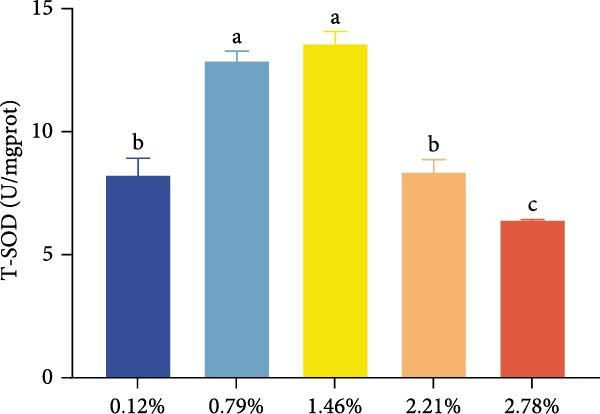
(B)

**Figure:**
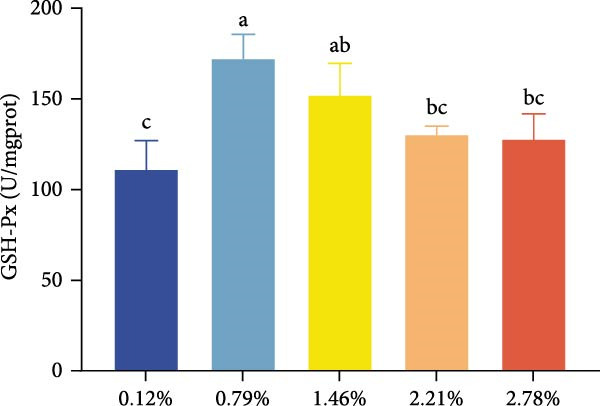
(C)

**Figure:**
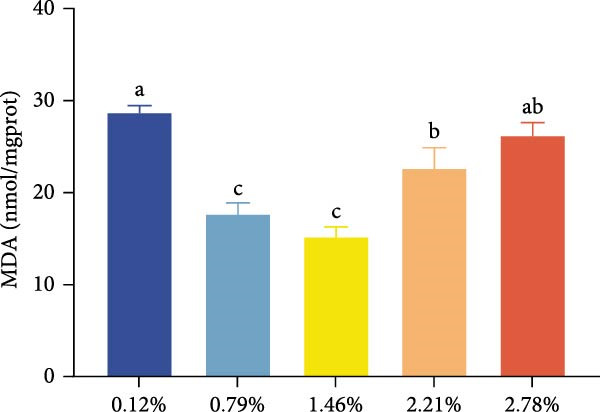
(D)

### 3.4. Effect of Different EPA Levels on the Ovarian Development in *M. rosenbergii* Broodstock

Analysis of ovarian development stages indicated that female prawns in the 1.46% EPA group exhibited well‐developed ovaries, with a high proportion reaching stages III and IV. In contrast, the 0.12% and 2.78% EPA groups retained a relatively large number of females at stages I and II. Correspondingly, the GSI was highest in the 1.46% EPA group, significantly exceeding that of the 0.12% and 2.78% groups (*p* < 0.05).

Dietary EPA levels also significantly influenced the secretion of steroid hormones. PROG content peaked in the 1.46% EPA group (*p*  < 0.05), while E2 exhibited a similar pattern, increasing initially and then decreasing with higher EPA levels, reaching its maximum in the third group (*p* < 0.05). Polynomial regression analysis indicated that dietary EPA levels of 1.32% and 1.5% could optimize the secretion of PROG and E2, respectively (Figure 2).

Figure 2Effect of different EPA levels on the ovarian development and steroid hormone in *M. rosenbergii* broodstock. (A) Proportion of ovarian developmental stages, (B) gonadosomatic index (GSI), and (C) progesterone (PROG). (D) Estradiol (E2), (E) polynomial regression analysis of progesterone, and (F) polynomial regression analysis of estradiol. Data are mean ± SD (*n* = 3). Different letters above bar indicate significant difference (*p* < 0.05).

**Figure:**
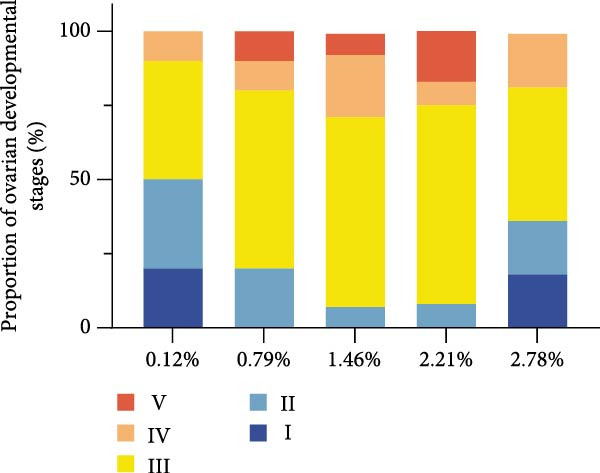
(A)

**Figure:**
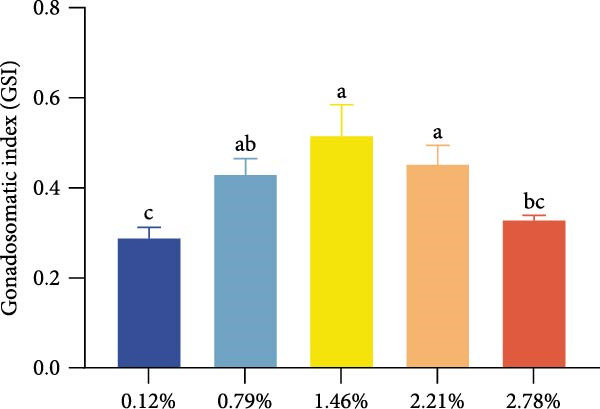
(B)

**Figure:**
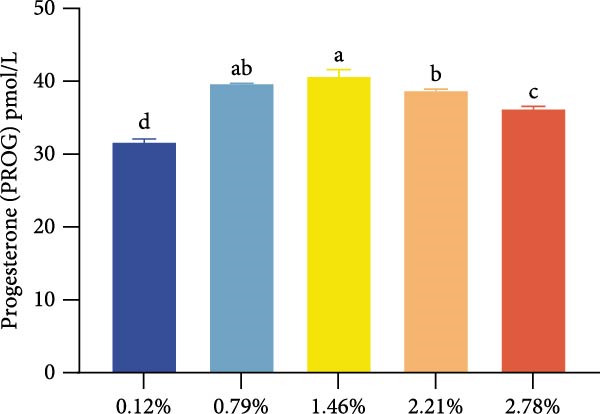
(C)

**Figure:**
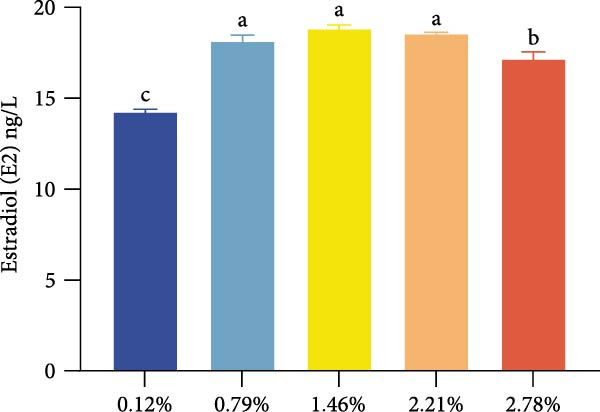
(D)

**Figure:**
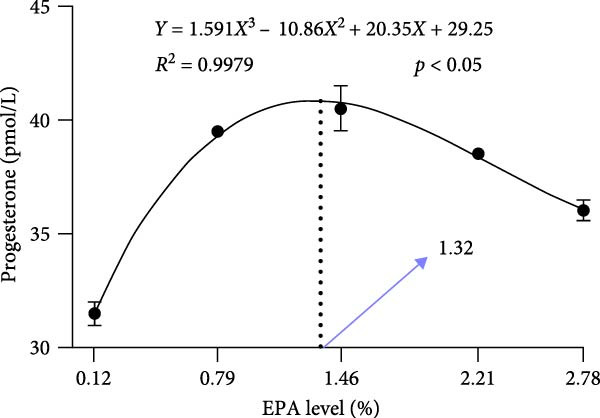
(E)

**Figure:**
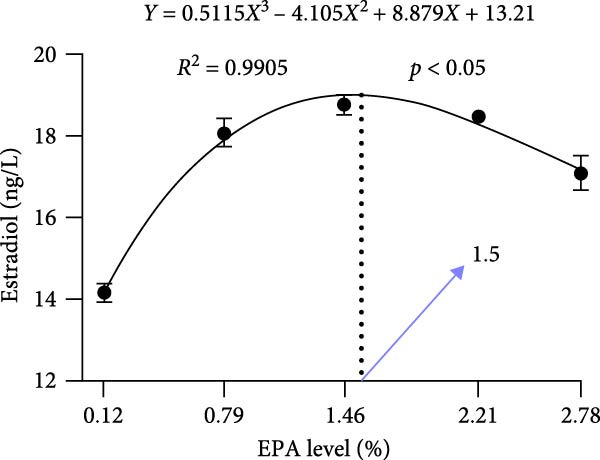
(F)

### 3.5. Effect of Different EPA Levels on the Expressions of Lipid Metabolism Genes in *M. rosenbergii* Broodstock

Different dietary EPA levels significantly influenced the expression of lipid metabolism‐related genes in the giant freshwater prawn broodstock (Figure 3). The expression levels of *fas*, *srebp-1*, and *scd* decreased progressively with increasing EPA concentration, reaching their lowest values in the 2.78% EPA group (*p* < 0.05). In contrast, the expression of *cpt-1* and *fabp-3* in the hepatopancreas initially increased and then declined with rising EPA levels (*p* < 0.05). A similar trend was observed for *acox-1*, although the differences among groups were not statistically significant.

Figure 3Gene expression of lipid metabolism in *M. rosenbergii* broodstock. (A) Fatty acid synthase (*fas*), (B) sterol regulatory element‐binding protein‐1 (*srebp-1*), (C) carnitine palmitoyl transferase‐1 (*cpt-1*), (D) acyl‐CoA oxidase‐1(*acox-1*), (E) fatty acid binding protein‐3 (*fabp-3*), and (F) stearoyl‐CoA desaturase (*scd*). Data are mean ± SD (*n* = 3). Different letters above bar indicate significant difference (*p* < 0.05).

**Figure:**
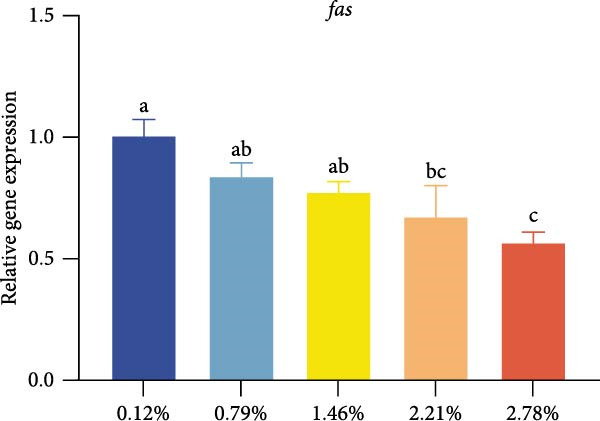
(A)

**Figure:**
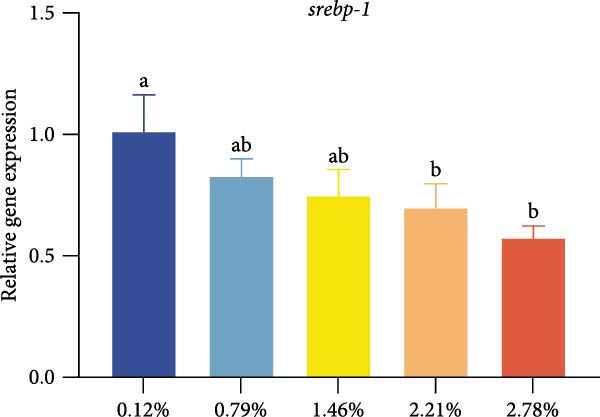
(B)

**Figure:**
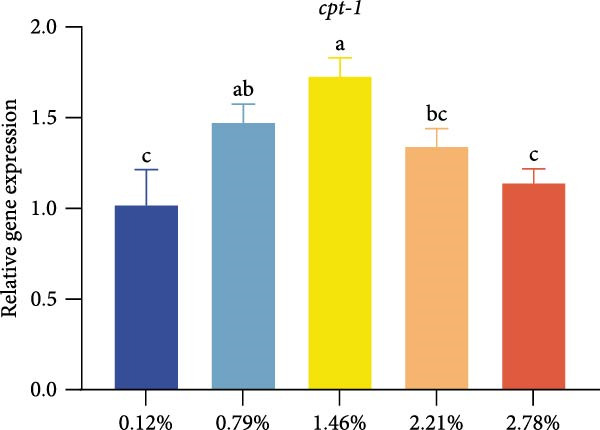
(C)

**Figure:**
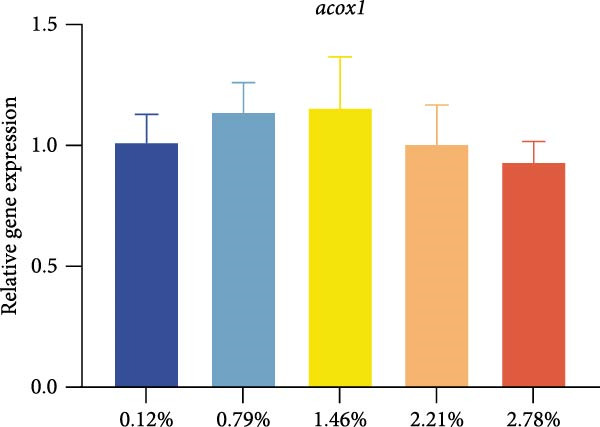
(D)

**Figure:**
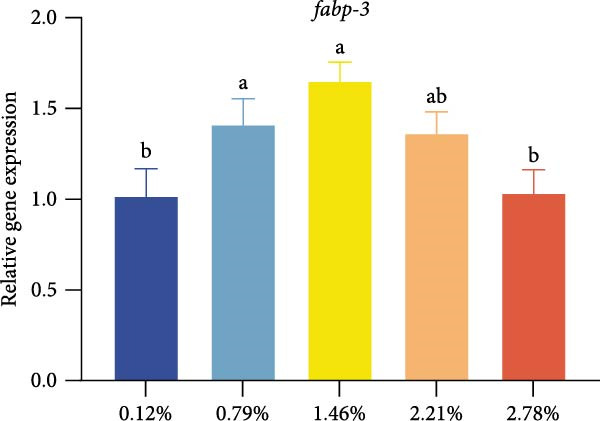
(E)

**Figure:**
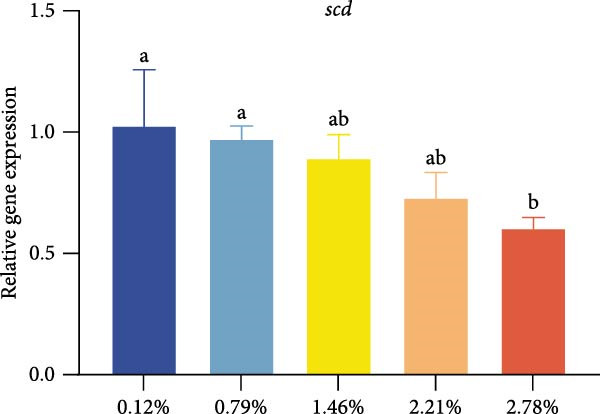
(F)

### 3.6. Effects of Different EPA Levels on the Hepatopancreatic and Ovarian Histological Structures of *M. rosenbergii* Broodstock

**Figure 4:**
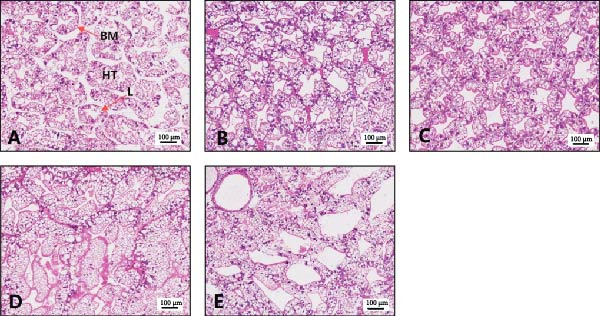
Section of hepatopancreas in female *M. rosenbergii* broodstock. (A) 0.12%EPA, (B) 0.79%EPA, (C) 1.46%EPA, (D) 2.21%EPA, and (E) 2.78%EPA. Scale bar represents 100 μm. BM, basement membrane; HT, hepatopancreatic tubules; L, lumen.

Histological observations revealed that dietary EPA levels markedly affected the hepatopancreatic tissue structure of the giant freshwater prawn broodstock (Figure 4). In the 0.79% and 1.46% EPA groups, hepatopancreatic tubules were well organized, with intact luminal structures, clear alignment, and uniformly thick basement membranes. By contrast, in the 0.12%, 2.21%, and 2.78% EPA groups, the tubules appeared disorganized, with loss of the characteristic star‐shaped lumen, more pronounced cellular vacuolation, and wider intertubular gaps.

**Figure 5:**
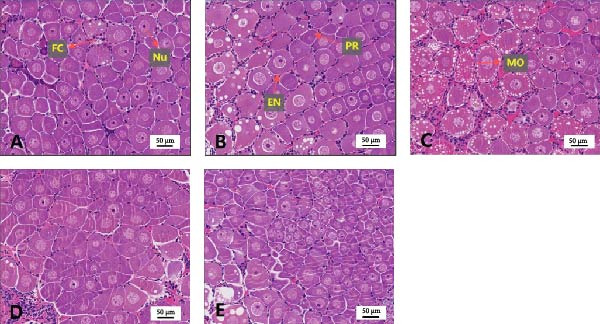
Section of the ovarian in female *M. rosenbergii* broodstock. (A) 0.12%EPA, (B) 0.79%EPA, (C) 1.46%EPA, (D) 2.21%EPA, and (E) 2.78%EPA. Scale bar represents 100 μ m. FC, follicular cells; Nu, nucleus; PR, previtellogenic oocytes; EN, endogenous vitellogenic oocytes; MO, mature oocytes.

Sections of the ovary from the giant freshwater prawn broodstock are shown in Figure 5. It can be observed that the number of mature oocytes in the 1.46% EPA group was significantly higher than that in other groups, while the 0.79% EPA group contained a large number of oocytes in the endogenous vitellogenesis stage, which were arranged closely. However, the oocytes in the 0.12%, 2.21%, and 2.78% EPA groups were more scattered than those in other groups, with lower cellular maturity.

## 4. Discussion

### 4.1. Effect of Different EPA Levels on the Growth Performance of *M. rosenbergii* Broodstock

The results indicated that dietary EPA levels had no significant effect on the survival rate of the giant freshwater prawn broodstock. A similar outcome was reported in studies examining the influence of PUFA proportions on the growth performance of prawn juveniles [19], suggesting that EPA exerts little effect on survival within a certain range. In the present study, the relatively low survival rate of broodstock may be attributed to environmental stressors or aggressive interactions during the rearing period. Nonetheless, the survival rate was consistent with observations from parallel aquaculture experiments and commercial production, thereby minimizing its impact on the validity of the results.

Previous research has demonstrated that dietary n‐3 HUFA can promote the growth of aquatic animals, although excessive levels may suppress growth and reproduction by inducing oxidative stress and impairing mitochondrial function [20, 21]. In this study, WG rate and SGR of broodstock initially increased with dietary EPA supplementation before declining, with the highest values observed in the 1.46% EPA group. Comparable patterns have been reported in coho salmon (*Oncorhynchus kisutch*) [22] and Pacific abalone (*Haliotis discus hannai*) [23], further supporting the notion that moderate EPA supplementation enhances growth performance, whereas excessive levels may be detrimental.

The reduced growth performance of prawn broodstock at low dietary EPA levels may be attributed to EPA deficiency, which has been associated with impaired immune function and growth retardation in aquatic organisms. Conversely, excessively high dietary EPA may induce immune stress, thereby inhibiting growth [24]. However, under the conditions of this experiment, differences in basic growth indicators such as WG and SGR were not statistically significant. This suggests that the physiological role of EPA in broodstock may be more pronounced in processes beyond general growth performance, particularly ovarian development.

In the present study, the hepatopancreatic index of the giant freshwater prawn broodstock decreased significantly with increasing dietary EPA levels. A similar phenomenon has been observed in juvenile black seabream (*Acanthopagrus schlegelii*), where diets enriched with different proportions of EPA and DHA reduced the HSI [25]. Previous studies have indicated that HUFA supplementation can lower HSI by decreasing hepatic lipid accumulation [26]. When considered alongside the expression profiles of lipid metabolism‐related genes, our results suggest that high dietary EPA concentrations may enhance fatty acid *β*‐oxidation and energy utilization in prawn. This metabolic shift likely reduces triglyceride deposition in the hepatopancreas [27], thereby contributing to the observed decrease in hepatopancreatic index.

### 4.2. Effect of Different EPA Levels on Fatty Acid Composition of Hepatopancreas in *M. rosenbergii* Broodstock

The dietary EPA level significantly influenced the fatty acid composition of the hepatopancreas in the giant freshwater prawn broodstock. Increasing EPA levels markedly elevated the contents of PUFA and HUFA, demonstrating that dietary fatty acid profiles directly shape hepatopancreatic fatty acid composition in prawn and exhibit a positive correlation [28]. This finding is consistent with previous reports. As a key n‐3 fatty acid, EPA can effectively enhance PUFA and HUFA deposition, which may result both from the enhanced conversion of C18 precursors (e.g., linolenic acid) mediated by *Δ*6 desaturase [29]. The synthesis of HUFA such as EPA also requires MUFA as substrates; accordingly, the MUFA content in the high‐EPA groups decreased significantly. Similar observations have been reported in other species, such as *Oplegnathus fasciatus*, where dietary DHA enrichment led to reduced MUFA levels [30]. Likewise, in a study on mice, high‐MUFA diets were shown to impair linolenic acid metabolism, thereby lowering its tissue content [31].

Because olive oil, which is rich in SFA, was used to adjust EPA levels in the diets, the SFA content of the feeds exhibited a decreasing trend. However, no significant differences in hepatopancreatic SFA content were detected among the broodstock groups. Previous studies have suggested that crustaceans preferentially metabolize SFA at different developmental stages, particularly during reproduction, while retaining PUFA to support essential functions such as membrane biosynthesis and reproduction [32]. This metabolic strategy may explain the relatively stable SFA levels in the hepatopancreas despite dietary variation. Interestingly, although the total SFA content remained unchanged, the proportion of stearic acid (C18:0) increased significantly across treatments. This may be linked to the suppression of *scd* gene expression, which encodes *scd*, the key enzyme responsible for converting C18:0 into oleic acid [33, 34]. Previous lipid metabolism studies have shown that EPA can downregulate *scd* [35], thereby reducing MUFA synthesis and leading to the accumulation of C18:0. We speculate that this shift may also be related to the incorporation of exogenous EPA into membrane phospholipids, which reduces the need for endogenous desaturation. Notably, while n‐3 fatty acids were significantly enriched with increasing dietary EPA levels, n‐6 fatty acids remained unaffected, suggesting that the metabolic pathways of n‐3 and n‐6 fatty acids operate relatively independently. The experimental results showed that the broodstock fed 2.78% EPA exhibited the highest hepatopancreatic HUFA levels; however, their ovarian development and antioxidant status were not optimal. This phenomenon is likely due to excessive EPA inducing oxidative stress and imposing a metabolic burden [36]. Although high‐dose EPA (2.78%) resulted in substantial deposition of HUFA in the hepatopancreas, its high degree of unsaturation renders membrane lipids more susceptible to peroxidation, which was directly reflected by the significant increase in MDA levels in this group, indicating aggravated oxidative damage [19]. Meanwhile, excessive EPA significantly downregulated genes related to lipid synthesis and transport, potentially disrupting normal lipid metabolic homeostasis and energy allocation. As a result, although the hepatopancreas accumulated abundant HUFA, it could not effectively transport them in an appropriate form or quantity to the ovaries for utilization; instead, the hepatopancreatic tissues exhibited observable structural damage, ultimately impairing their normal physiological functions and leading to hindered ovarian development and reduced overall antioxidant capacity.

### 4.3. Effect of Different EPA Levels on Antioxidant Capacity in *M. rosenbergii* Broodstock

During metabolism, crustaceans generate large amounts of free radicals, and excessive accumulation can lead to oxidative stress, which negatively impacts normal growth and development. The antioxidant capacity of an organism primarily depends on the activities of enzymatic antioxidants such as T‐AOC, T‐SOD, and GSH‐Px [37]. Meanwhile, MDA, a major marker of lipid peroxidation, serves as an indicator of oxidative damage; its reduction reflects improved cell membrane integrity and function, thereby supporting normal hepatopancreatic metabolism [19]. Thus, when assessing the antioxidant performance of prawns, it is essential to combine enzymatic antioxidant activities with MDA levels for a comprehensive evaluation.

In this study, dietary supplementation with 0.79%–1.46% EPA significantly enhanced the antioxidant performance of the giant freshwater prawn broodstock. Previous research has suggested that PUFA can exert antioxidant effects by modulating redox‐related signaling pathways [38, 39]. Accordingly, we infer that appropriate EPA levels help mitigate free radical‐induced lipid damage, reinforce antioxidant defenses, and reduce the formation of lipid peroxides. EPA may also alleviate oxidative stress by competitively inhibiting the synthesis of pro‐inflammatory metabolites derived from n‐6 PUFA [29]. However, at higher dietary levels, EPA caused a significant increase in MDA content and a decline in antioxidant capacity. This aligns with evidence that excessive n‐3 PUFA incorporation into membranes heightens susceptibility to lipid peroxidation compared with n‐6 PUFA [40]. Such imbalance can induce excessive inflammatory responses, aggravate oxidative stress, and ultimately compromise hepatopancreatic structure and function in broodstock prawn. Nonetheless, some studies in fish have reported no significant effects of dietary EPA on antioxidant indices [24], highlighting the need for further research to elucidate the underlying mechanisms by which HUFA regulates oxidative balance in aquatic animals.

### 4.4. Effect of Different EPA Levels on the Ovarian Development in *M. rosenbergii* Broodstock

The results showed that the GSI of female prawns in the 1.46% EPA group reached the highest level, which was consistent with the group exhibiting higher ovarian maturity. Moreover, a large number of mature oocytes were observed in the ovarian sections of this group. Histological sections further confirmed this, revealing a large number of mature oocytes in this group. Similar findings have been reported in Chinese mitten crab (*Eriocheir sinensis*) broodstock, where HUFA‐enriched diets promoted ovarian development in crustaceans [41]. These results suggest that an appropriate level of dietary EPA facilitates nutrient transfer from the hepatopancreas to the ovary, thereby increasing ovarian weight, elevating the GSI, and accelerating ovarian development.

Beyond its direct role in oocyte maturation, EPA may also support reproductive health by modulating hormone synthesis and secretion. Studies in mammals have shown that EPA supplementation elevates luteinizing hormone and T levels, increases follicular cell layer thickness, and accelerates oocyte maturation [42]. In the present study, both PROG and E2 levels increased with dietary EPA concentration, peaking in the 1.46% group. This suggests that EPA may influence sex hormone synthesis through similar mechanisms. Therefore, we speculate that the positive effects of dietary EPA supplementation on ovarian development in *M. rosenbergii* broodstock may also be related to the regulation of hormone secretion by EPA levels. Moreover, EPA can regulate cell membrane fluidity and cholesterol metabolism, thereby modulating hormone receptor activity and secretion [43, 44]. In synergy with steroid hormones such as PROG and E2, this mechanism may further promote oocyte maturation and ovarian function.

However, excessive dietary EPA exerted inhibitory effects on ovarian development. For instance, in olive flounder (*Paralichthys olivaceus*) broodstock, excessive HUFA supplementation reduced sex hormone secretion and reproductive performance [45]. A possible explanation is that high EPA levels induce oxidative stress or disrupt lipid metabolic homeostasis, thereby lowering hormone secretion and impairing oocyte maturation.

In summary, moderate dietary EPA supplementation significantly enhanced steroid hormone secretion and GSI in the giant freshwater prawn, promoting ovarian development. In contrast, both insufficient and excessive levels were unfavorable to ovarian function. Multiple regression analysis indicated that dietary EPA levels of ~1.32% and 1.50% were optimal for stimulating PROG and E2 secretion, respectively. The relatively high demand for EPA during ovarian development may reflect the increased requirement for HUFA to support reproduction and nutrient allocation during the breeding period, particularly under conditions with a balanced n‐3/n‐6 ratio. Future studies should further investigate the molecular mechanisms through which EPA regulates ovarian development and explore the combined effects of other n‐3 HUFA and fatty acid ratios, with the aim of developing optimized nutritional strategies for prawn broodstock.

### 4.5. Effects of Different EPA Levels on Lipid Metabolism in *M. rosenbergii* Broodstock

The hepatopancreas is a vital organ for lipid storage and metabolism, and it can transport lipids to the gonads via hemolymph to support ovarian development. Therefore, lipid metabolism indicators in the hepatopancreas are also among the key aspects to focus on when studying broodstock development. *Fas* generates long‐chain fatty acids through the reaction of acetyl‐CoA and malonyl‐CoA, and it is a key enzyme gene in the process of fatty acid biosynthesis. Changes in its activity and expression levels directly reflect the fat synthesis process in animals [46, 47]. Notably, the structure and position of fatty acid carbon chains have a significant effect on *fas* activity; for example, n‐3 series fatty acids are effective inhibitors of *fas* [48]. At the same time, *fas* is also regulated by the transcription factor *srebp-1* during de novo fatty acid synthesis [49]. The results of this study showed that with increasing dietary EPA levels, the relative expression of *srebp-1* and *fas* genes in the hepatopancreas of the giant freshwater prawn broodstock showed a downward trend. This indicates that the expression of lipid synthesis‐related genes in prawn is regulated by dietary EPA, thereby affectinglipid metabolism in the hepatopancreas and leading to a significant decrease in the HSI. The expression of lipid decomposition‐related genes was similar to the findings of Lee et al. [50], namely, EPA significantly upregulated the expression of the *cpt-1* gene while downregulating the expression of genes involved in lipogenesis. In addition, the insignificant effect of EPA on *acox-1* expression may be related to the fact that crustaceans preferentially oxidize EPA via the mitochondrial rather than the peroxisomal pathway.

Lipid metabolism is a complex process involving fatty acid synthesis, degradation, and transport. As a key mediator of lipid signaling, *fabp* specifically binds acyl molecules and mediates their directional transport to subcellular structures such as mitochondria, peroxisomes, and the nucleus, thereby participating in both lipid catabolism and biosynthesis [51]. Through a ligand–receptor interaction mechanism, this protein family accurately guides fatty acid precursors into the mitochondrial matrix for *β*‐oxidation or targets them to the endoplasmic reticulum lipid synthesis compartment to generate triglycerides and phospholipids, thus effectively regulating lipid homeostasis [52]. Previous studies have shown that the level of long‐chain fatty acids in food is positively correlated with *fabp* expression, suggesting that long‐chain fatty acids have an inductive effect on *fabp* [53]. Therefore, in this study, dietary EPA promoted the upregulation of *fabp* gene expression; however, when EPA levels exceeded a certain range, this promoting effect began to decline. At the same time, *scd* expression in the hepatopancreas was significantly reduced in all treatment groups, This result was consistent with the findings of [54], who reported that in mice fed diets containing primarily EPA fats, expression of the hepatic *scd1* message was markedly suppressed. Thus, we suggest that *scd* activity in the hepatopancreas is associated with dietary EPA levels. Studies have confirmed that EPA can influence lipid synthesis and degradation by regulating transcription factors and gene expression [55]. On the one hand, EPA can inhibit the expression of fatty acid synthesis‐related genes by affecting upstream transcription factors such as *srebp-1* and promote lipid transport and catabolism. On the other hand, excessively high levels of EPA may suppress the expression of these genes, thereby affecting lipid metabolic balance.

## 5. Conclusion

In summary, dietary supplementation with 1.46% EPA optimally supports the broodstock performance of the giant freshwater prawn. At this level, EPA promotes growth, enhances antioxidant capacity, and stimulates steroid hormone secretion, thereby facilitating oocyte maturation and ovarian development. Furthermore, an appropriate EPA supply contributes to energy provision by enhancing fatty acid transport and mitochondrial *β*‐oxidation. These findings highlight the critical role of balanced dietary EPA in improving broodstock quality. In summary, dietary supplementation with 1.46% EPA optimally supports the broodstock performance of the giant freshwater prawn. Therefore, this study recommends maintaining EPA levels within this range in practical broodstock feed formulation to effectively enhance ovarian development and overall health status.

## Conflicts of Interest

The authors declare no conflicts of interest.

## Author Contributions

Yuxin Sun and Hao Chen contributed equally to this work.

## Funding

This work was supported by “Pioneer” and “Leading Goose” R&D Program of Zhejiang (2023C02024).

## Data Availability

The data that support the findings of this study are available from the corresponding author upon reasonable request.
